# Rare Orbital Metastasis of Carcinoid Tumor Despite Long-Term Somatostatin Therapy: A Case Report

**DOI:** 10.3390/reports8030158

**Published:** 2025-08-28

**Authors:** Hritika Hosalkar, Leo Meller, Nahia Dib El Jalbout, Marissa K. Shoji, Sally L. Baxter, Don O. Kikkawa

**Affiliations:** 1 Division of Oculofacial Plastic and Reconstructive Surgery, Viterbi Family Department of Ophthalmology, UC San Diego Shiley Eye Institute, La Jolla, CA 92037, USA; 2 Division of Biomedical Informatics, Department of Medicine, UC San Diego School of Medicine, La Jolla, CA 92037, USA; 3 Division of Plastic Surgery, UC San Diego School of Medicine, La Jolla, CA 92037, USA

**Keywords:** carcinoid tumor, metastasis, orbit, somatostatin, case report

## Abstract

**Background and Clinical Significance**: Carcinoid tumors are rare, slow-growing neuroendocrine cell neoplasms that typically affect the gastrointestinal tract. While metastasis may occur, it most commonly occurs in the liver, and orbital metastasis is extremely rare, especially while on systemic somatostatin suppression. **Case Presentation**: A 57-year-old man with a history of gastrointestinal carcinoid tumor treated with lanreotide for 5 years presented with a left proptotic, red eye and double vision for several months. Clinical examination revealed left proptosis, supraduction deficit, lower lid retraction, and dilated episcleral vessels inferiorly. Magnetic resonance imaging demonstrated a 1.8 cm enhancing lesion centered within the left inferior rectus muscle. Left orbitotomy and biopsy were performed, which confirmed metastatic carcinoid tumor. He will undergo localized orbital radiation and substitution of lanreotide with systemic chemotherapy. **Conclusions**: Orbital metastasis of carcinoid tumor is extremely uncommon. Given its rarity, diagnosis may be challenging. In patients presenting with ocular complaints including chronic red eye, double vision, proptosis, and mass effect with a prior history of neuroendocrine cancer, a high index of suspicion for orbital metastasis is necessary with timely workup and treatment even if the disease has been otherwise well-controlled with somatostatin analogs.

## 1. Introduction and Clinical Significance

Carcinoid tumors, a subset of well-differentiated neuroendocrine neoplasms, are characterized by their indolent behavior and potential for hormone secretion [1]. The prevalence of carcinoid tumors has been estimated to be 9.2/100,000 individuals [2], with the most common primary sites including the gastrointestinal tract, particularly the small intestine and rectum, and the bronchopulmonary system [3]. Although carcinoid tumors are typically well-differentiated, they can metastasize, with common affected sites being the liver, lung, bone, and brain [3].

Orbital metastasis from carcinoid tumor has been described but is rare. Additionally, orbital metastasis in the setting of ongoing long-term systemic disease suppression with somatostatin analogs is exceedingly uncommon. Herein, we report a case of orbital metastasis from a carcinoid tumor in a 57-year-old man with a stage IV gastrointestinal carcinoid tumor who had been continuously treated with lanreotide for 5 years prior to orbital presentation. This report aims to characterize the unique presentation and challenges in the diagnosis, workup, and management of this rare clinical entity.

## 2. Case Presentation

**Figure 1:**
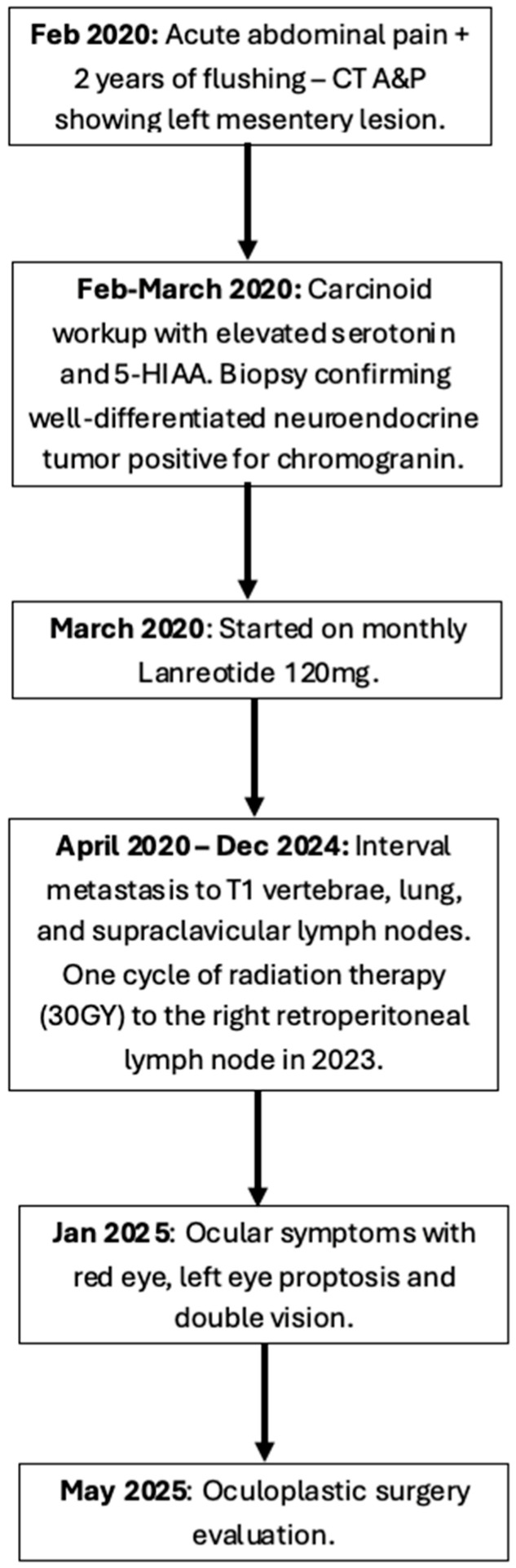
**Oncologic history timeline.** CT A&P: computerized tomography of the abdomen and pelvis.

A 57-year-old man was referred to oculoplastic surgery for chronic red eye, double vision, and left eye proptosis for four months. His past medical history included a neuroendocrine tumor of small bowel origin that was diagnosed five years prior, and a visualization of the patient’s oncologic history is provided in Figure 1. The patient presented at that time with acute abdominal pain and flushing and underwent imaging of the abdomen and pelvis that demonstrated a 2.7 cm left mesenteric lesion with retroperitoneal lymphadenopathy. The workup was notable for elevated serum serotonin levels (939 ng/mL, normal: 50–200 ng/mL), and elevated plasma 5-HIAA levels (77 ng/mL, normal 0–20 ng/mL) with computerized tomography (CT)-guided core biopsy confirming a well-differentiated neuroendocrine tumor positive for chromogranin. He was started on monthly injections of lanreotide 120 mg and received one cycle of radiation therapy (30GY) to the right retroperitoneal lymph node. In the five years between primary carcinoid presentation and ocular symptoms, other metastatic lesions were found in the T1 vertebrae, lung, and supraclavicular lymph nodes. However, these were observed with serial CT imaging, and given the stability of these lesions, he remained on lanreotide alone without evidence of further local progression. Of note: A routine Positron Emission Tomography (PET)/Computed Tomography (CT) was not performed given that localized CT scans of known lesions showed stable disease, and no orbital involvement was previously clinically noted.

**Figure 2:**
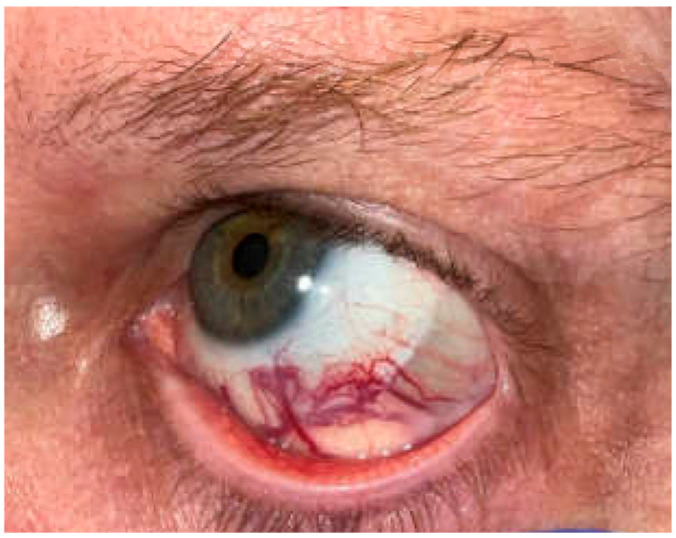
**Clinical photos of the patient at his initial presentation to oculoplastic surgery.** On exam, a palpebral conjunctival lesion with dilated vessels was noted at the left inferior bulbar, concerning orbital extension of a systemic carcinoid tumor.

**Figure 3:**
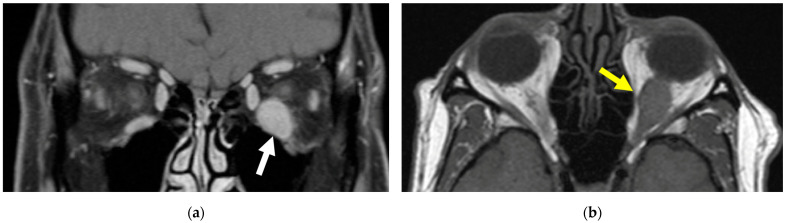
**MRI orbit with and without contrast.** An enlarging 18 mm enhancing lesion (**white arrow**, (**a**)) is present and centered within the left inferior rectus muscle (**yellow arrow**, (**b**)).

On exam at the time of his presentation for oculoplastic surgery, a left inferior bulbar and palpebral conjunctival lesion was noted with surrounding dilated vessels and concern for orbital extension (Figure 2). Left lower lid retraction, 2 mm of left proptosis, and a supraduction deficit of the left eye were also present, although there was no evidence of optic nerve compromise. Magnetic resonance imaging (MRI) of the orbit (Figure 3) showed an enlarging 18 mm enhancing lesion centered within the left inferior rectus muscle.

**Figure 4:**
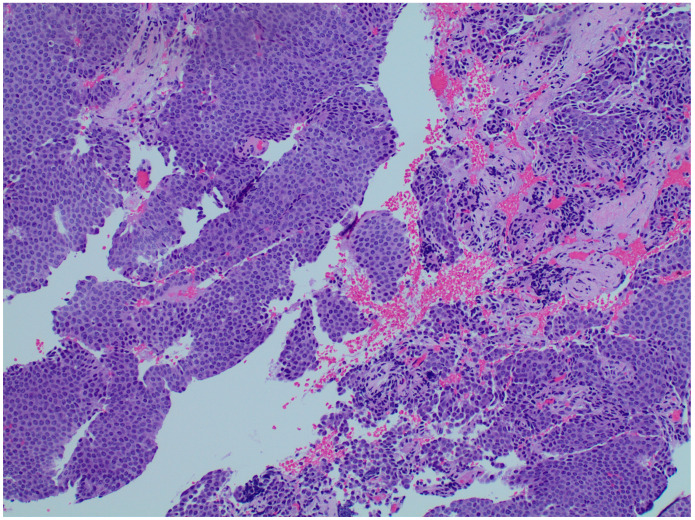
**Orbital biopsy histopathology (magnification, 100×).** Orbital biopsy demonstrates nests and lobules of cells with uniform round nuclei and evenly dispersed, speckled chromatin invading a background of fibrovascular tissue, consistent with a well-differentiated neuroendocrine tumor.

**Figure 5:**
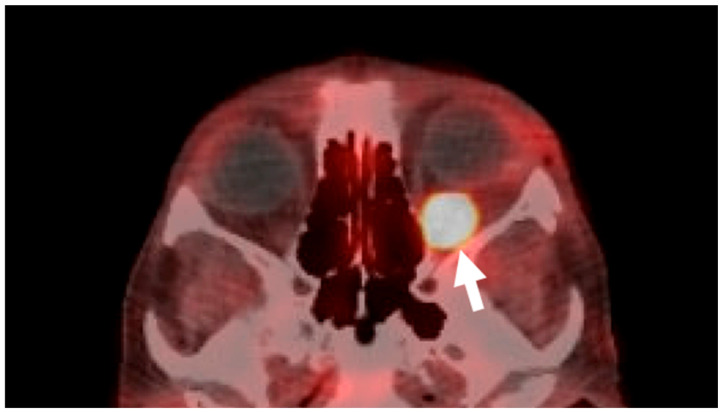
**PET/CT Cu64 Dotatate scan.** An axial view of the orbits demonstrates new focal uptake in the left retrobulbar region (**arrow**) is present, involving the left inferior rectus muscle.

Given the concern for malignancy, especially due to the patient’s prior oncologic history, an orbitotomy with biopsy was performed with results confirming a neuroendocrine carcinoid tumor metastatic to the orbit (Figure 4). A PET/CT Cu64 Dotatate scan specific for neuroendocrine tumors was performed and demonstrated new focal uptake in the left retro-orbital region (Figure 5) as well as new bone metastasis involving the humeri, spine, pelvis, ribs, sternum, scapulae, new hepatic lesions, and new bilateral scrotum lesions. The patient was referred to radiation oncology for orbital radiation as well as oncology with the plan to stop lanreotide and initiate systemic chemotherapy.

## 3. Discussion

This case highlights a unique presentation of orbital metastasis of a systemic carcinoid tumor despite long-term suppression with somatostatin therapy. While orbital carcinoid metastasis has been described in the literature, primarily in case reports and smaller case series [4,5,6,7,8,9], breakthrough metastasis despite systemic suppression is exceedingly rare. Only a few reports have been published, one of which involved a primary renal carcinoid tumor metastatic to the orbit with left lateral rectus involvement; however, this patient had only received octreotide and everolimus chemotherapy for less than one year before orbital metastasis [10]. In that case, the patient maintained stable proptosis with no changes in vision or diplopia, and thus no interventions specific for orbital metastasis were pursued. Peixoto et al. also reported a 79-year-old woman with a carcinoid tumor of small bowel origin with liver metastasis and without carcinoid syndrome who developed orbital metastasis with proptosis despite being on octreotide for two years [11]. She was treated with a four-week course of orbital radiation with size reduction of the lesion and improving diplopia. In our case, localized CT scans of known lesions, rather than systemic PET/CT, were used to monitor carcinoid disease progression prior to orbital metastasis, which is consistent with National Comprehensive Cancer Network (NCCN) guidelines [12]. Therefore, although it is challenging to determine the exact timing of orbital metastasis, the new onset of ocular symptoms five years after systemic somatostatin suppression likely approximates the time of orbital spread. To the best of our knowledge, the five-year interval in our case represents the longest reported duration of systemic disease suppression prior to orbital metastasis of a carcinoid tumor.

Orbital carcinoid tumors are extremely rare; as such, studies assessing clinical characteristics and outcomes are often limited. In one of the largest published studies, 28 patients with carcinoid tumors metastatic to the orbit were analyzed across eight tertiary academic centers [13]. Half of patients (14) had primary tumors of gastrointestinal origin, followed by lung (7.1%, two patients), kidney (7.1%, two patients), with two patients (7.1%) presenting with localized orbital disease. The mean age at diagnosis of the primary tumor was 58.8 years, with a mean age of orbital disease at 62.6 years, with an average interval time from primary diagnosis to orbital metastasis of 4.4 years. The most common presenting symptoms were proptosis (71%) and diplopia (61%), with extraocular muscle involvement noted in 79% of patients. Management varied widely across the cohort, with surgery being the most common treatment modality (61%), followed by octreotide (39%), radiation (36%), and chemotherapy (32%). The most common surgery performed was debulking (10/28, 35.7%), followed by en bloc resection and localized resection (both 3/28, 10.7%), then exenteration (2/28, 7.1%). Importantly, unilateral orbital disease was associated with longer progression-free survival and time to death, and survival did not differ according to treatment modality among surgery, radiation, and octreotide. Survival also did not differ between incisional (debulking) and excisional (resection or exenteration) surgeries. Primary carcinoid disease of the orbit was associated with longer time to death from all causes when compared to orbital metastasis of systemic disease. Chemotherapy was associated with shorter survival time; however, this may have been confounded by patients with higher disease burden being selected for the treatment. Importantly, the 5-year survival rate was reported as 81.8% from diagnosis of primary tumor and a 50% survival rate from diagnosis of orbital metastasis, which highlights the critical need for prompt workup and treatment when orbital involvement is suspected. Interestingly, Mustak et al. did not specify whether any orbital metastasis occurred while patients were on systemic suppression with somatostatin analogs. Hence, the true prevalence and clinical characteristics of orbital metastasis despite ongoing somatostatin suppression remain unknown.

Diagnosis of orbital metastasis of carcinoid tumors generally relies on a thorough clinical history and imaging evaluation, with orbital biopsy providing definitive confirmation. One key imaging scan for carcinoid disease detection is PET/CT with Cu64 Dotatate. This was utilized in our patient and has one of the highest sensitivity (100%) and specificity (96.8%) as reported by Delpassand et al. in their prospective clinical trial in 2020 [14]. While there are nuances in treatment as described above, somatostatin analogs may be useful for both reducing carcinoid tumor hormonal hypersecretion as well as their antiproliferative effects to inhibit tumor growth. In terms of management, our patient was on lanreotide instead of octreotide. Both lanreotide and octreotide are considered appropriate interventions by the NCCN to reduce carcinoid disease progression, but lanreotide has been associated with ease of administration given its subcutaneous rather than intramuscular formulation as well as higher patient satisfaction [15]. Extensive surgical resection was not pursued for our patient given there were no clear mortality benefits with surgery as noted above and potential morbidity given the size and location of the tumor, and thus orbital radiation in combination with systemic chemotherapy was planned for our patient given his advanced disease with new multifocal systemic involvement.

Overall, orbital metastasis of carcinoid tumors in the setting of long-term systemic suppression is extremely rare. Possible reasons behind such occurrences include heterogeneous tumor characteristics (varying somatostatin receptor expression levels that influence treatment effectiveness) [16] and treatment resistance related to receptor internalization, sensitization, and complex tumor microenvironment [17]. While the exact mechanism in our case remains elusive and our report is limited by the lack of long-term follow up, our case highlights that physicians should remain vigilant about the risk of metastasis despite long-term systemic suppression, especially considering that orbital metastasis confers a significantly lower survival rate [13]. Appropriate and prompt workup for metastasis should be initiated to optimize patient treatment and outcomes.

## 4. Conclusions

Orbital metastasis of systemic carcinoid tumor is rare. Clinicians should remain aware that late metastasis may still occur despite disease stabilization with somatostatin analog, and a high index of suspicion should be maintained for orbital metastasis to facilitate timely workup and treatment.

## Data Availability

The original contributions presented in this study are included in the article. Further inquiries can be directed at the corresponding author.
